# Sinomenine, a natural dextrorotatory morphinan analog, is anti-inflammatory and neuroprotective through inhibition of microglial NADPH oxidase

**DOI:** 10.1186/1742-2094-4-23

**Published:** 2007-09-19

**Authors:** Li Qian, Zongli Xu, Wei Zhang, Belinda Wilson, Jau-Shyong Hong, Patrick M Flood

**Affiliations:** 1 Comprehensive Center for Inflammatory Disorders, University of North Carolina, Chapel Hill, North Carolina 27599, USA; 2 Neuropharmacology Section, National Institute of Environmental Health Sciences, National Institutes of Health, Research Triangle Park, North Carolina 27709, USA; 3 Epidemiology branch, National Institute of Environmental Health Sciences, National Institutes of Health, Research Triangle Park, North Carolina 27709, USA

## Abstract

**Background:**

The mechanisms involved in the induction and regulation of inflammation resulting in dopaminergic (DA) neurotoxicity in Parkinson's disease (PD) are complex and incompletely understood. Microglia-mediated inflammation has recently been implicated as a critical mechanism responsible for progressive neurodegeneration.

**Methods:**

Mesencephalic neuron-glia cultures and reconstituted cultures were used to investigate the molecular mechanisms of sinomenine (SN)-mediated anti-inflammatory and neuroprotective effects in both the lipopolysaccharide (LPS)- and the 1-methyl-4-phenylpyridinium (MPP^+^)-mediated models of PD.

**Results:**

SN showed equivalent efficacy in protecting against DA neuron death in rat midbrain neuron-glial cultures at both micro- and sub-picomolar concentrations, but no protection was seen at nanomolar concentrations. The neuroprotective effect of SN was attributed to inhibition of microglial activation, since SN significantly decreased tumor necrosis factor-α (TNF-α, prostaglandin E_2 _(PGE_2_) and reactive oxygen species (ROS) production by microglia. In addition, from the therapeutic point of view, we focused on sub-picomolar concentration of SN for further mechanistic studies. We found that 10^-14 ^M of SN failed to protect DA neurons against MPP^+^-induced toxicity in the absence of microglia. More importantly, SN failed to show a protective effect in neuron-glia cultures from mice lacking functional NADPH oxidase (PHOX), a key enzyme for extracellular superoxide production in immune cells. Furthermore, we demonstrated that SN reduced LPS-induced extracellular ROS production through the inhibition of the PHOX cytosolic subunit p47^*phox*^translocation to the cell membrane.

**Conclusion:**

Our findings strongly suggest that the protective effects of SN are most likely mediated through the inhibition of microglial PHOX activity. These findings suggest a novel therapy to treat inflammation-mediated neurodegenerative diseases.

## Background

Increasing evidence has shown that the production and accumulation of pro-inflammatory and cytotoxic factors by over-activated microglia are closely associated with the pathogenesis of several neurological disorders, such as Parkinson's disease (PD), Alzheimer's disease, amyotrophic lateral sclerosis, multiple sclerosis, and the AIDS dementia complex [1-4]. Microglia are the main immune cells in the brain, which provides the CNS immune protection against infection under normal physiologic conditions [5]. However, in pathological conditions microglia may easily become over-activated and produce large amounts of pro-inflammatory factors, such as cytokines, prostaglandins, and reactive oxygen species (ROS), which result in neuronal death [6-8]. Microglia are found in great abundance in the midbrain region that encompasses the substantia nigra [9]. Thus, it has been suggested that activation of nigral microglia and subsequent release of these pro-inflammatory neurotoxic factors is a critical component of the degenerative process of dopaminergic (DA) neurons in PD. Unfortunately, therapies using specific inhibitors to prevent the production of individual pro-inflammatory and neurotoxic factors have not been particularly successful, suggesting that the mechanism of inflammation-mediated damage to DA neurons has yet to be elucidated. In addition, current DA replacement therapy for PD is only able to treat the symptoms, and fails to slow down the progression of the disease. Therefore, there is an urgent need to develop drugs that have wide spectrum anti-inflammatory effects and which are able to slow down or curtail the progression of the degenerative process.

The alkaloid sinomenine (SN) is a pure compound extracted from the Chinese medicinal plant, *sinomenium acutum*, which has been utilized to treat inflammatory diseases for many centuries [10,11]. Clinical trials have demonstrated that purified SN has significant therapeutic efficacy for patients who suffer from rheumatoid arthritis [10,12]. Previous pharmacological studies have demonstrated that the pharmacological profile of SN includes immunosuppression [13], arthritis amelioration [14], anti-inflammation [15] and protection against hepatitis induced by lipopolysaccharide (LPS) [16]. In addition, in studies using intramuscular injection and multiple dosing, a combination of SN and cyclosporin A showed immunomodulatory effects in a cardiac transplant model [17]. Up to now, little is known about the molecular mechanism by which SN exhibits immunomodulatory effects.

Based on its molecular structure, SN belongs to the family of morphinans. Morphinans are a series of compounds structurally similar to morphine, but lacking the E ring as well as the 6-OH and the 7, 8-double bond. Our previous studies have clearly shown that several morphinan compounds, including naloxone [18] and dextromethorphan [19], are neuroprotective anti-inflammatory agents. Given the reported anti-inflammatory properties of SN, the record of its effective use in clinical therapy, its natural abundance, and its relative low cost in purification and preparation, we theorized that this compound might be a better alternative than synthetic morphinan compounds in inhibiting inflammatory destruction of DA neurons. Moreover, the finding that it is a naturally occurring dextrorotatory morphinan isomer indicates that it would have minimal interaction with opiate receptors, and therefore prove to be a much safer alternative than some other morphinan compounds when used chronically to treat PD. In this study, we report that SN shows a significant neuroprotective effects against both LPS- and MPP^+^-induced DA neurotoxicity, and that this protection is mediated through microglia. The finding that SN is acting to inhibit NADPH oxidase (PHOX) activity, which then results in the inhibition of a wide array of pro-inflammatory mediators produced by activated microglia, suggests that SN may be a potential novel and safe therapeutic agent for the treatment of inflammatory-mediated neurodegenerative diseases.

## Methods

### Animals

PHOX-deficient (gp91phox^-/-^) and wild-type C57BL/6J (gp91phox^+/+^) mice were obtained from The Jackson Laboratory (Bar Harbor, ME). Breeding of the mice was performed to achieve timed pregnancy with the accuracy of ± 0.5 days. Timed-pregnant Fisher F344 rats were obtained from Charles River Laboratories (Raleigh, NC). Housing and breeding of the animals were performed in strict accordance with the National Institutes of Health guidelines.

### Reagents

SN was obtained from Sigma-Aldrich (St. Louis, MO). LPS (strain O111:B4) was purchased from Calbiochem (San Diego, CA). Cell culture reagents were obtained from Invitrogen (Carlsbad, CA). [^3^H]-DA (30 Ci/mmol) was obtained from Perkin-Elmer Life Sciences (Boston, MA), and the polyclonal anti-tyrosine hydroxylase antibody was a generous gift from Dr. John Reinhard (GlaxoSmithKline, Research Triangle Park, NC). The Vectastain ABC kit and biotinylated secondary antibodies were purchased from Vector Laboratories (Burlingame, CA). The fluorescence probe Dichlorodihydro-fluorescein Diacetate (DCFH-DA) was obtained from Calbiochem (La Jolla, CA).

### Microglial cell line

The rat microglia HAPI cell line was a generous gift from Dr. James R. Connor [20]. Briefly, cells were maintained at 37°C in DMEM supplemented with 10% FBS, 50 U/ml penicillin, and 50 μg/ml streptomycin in a humidified incubator with 5% CO2 and 95% air.

### Primary mesencephalic neuron-glia culture

Neuron-glia cultures were prepared from the ventral mesencephalic tissues of embryonic day 14–15 rats or day 13–14 mice, as described previously [18]. Briefly, dissociated cells were seeded at 1 × 10^5^/well and 5 × 10^5^/well to poly-D-lysine-coated 96-well and 24-well plates respectively. Cells were maintained at 37°C in a humidified atmosphere of 5% CO_2 _and 95% air, in MEM containing 10% fetal bovine serum, 10% horse serum, 1 g/L glucose, 2 mM L-glutamine, 1 mM sodium pyruvate, 100 μM nonessential amino acids, 50 U/ml penicillin, and 50 μg/ml streptomycin. Seven-day-old cultures were used for treatment. At the time of treatment, immunocytochemical analysis indicated that the rat neuron-glia cultures were made up of 11% microglia, 48% astrocytes, 41% neurons, and 1% tyrosine hydroxylase immunoreacitve (TH-IR) neurons. The composition of the neuron-glia cultures of PHOX-deficient mice was very similar to that of the wild-type mice consisting of 12% microglia, 48% astrocytes, 40% neurons, and 1% TH-IR neurons.

### Primary mesencephalic neuron-enriched and microglia-enriched Cultures

Midbrain neuron-enriched cultures were established as described previously [21]. Briefly, 24 h after seeding the cells, cytosine β-D-arabinocide was added to a final concentration of 5 μM to suppress glial proliferation. Three days later, cultures were changed back to maintenance medium and were used for treatment 7 days after initial seeding. Rat microglia-enriched cultures, with a purity of > 98%, were prepared from whole brains of 1-day-old Fischer 344 rat pups as described previously [21].

### [^3^H]-DA uptake assay

[^3^H]-DA uptake assays were performed as described [22]. Briefly, cells were incubated for 20 min at 37°C with 1 μM [^3^H]-DA in Krebs-Ringer buffer (16 mM sodium phosphate, 119 mM NaCl, 4.7 mM KCl, 1.8 mM CaCl_2_, 1.2 mM MgSO_4_, 1.3 mM EDTA, and 5.6 mM glucose; pH 7.4). After washing three times with ice-cold Krebs-Ringer buffer, the cells were collected in 1 N NaOH. Radioactivity was determined by liquid scintillation counting. Nonspecific DA uptake observed in the presence of mazindol (10 μM) was subtracted.

### Immunostaining

DA neurons were recognized with the anti-TH antibody as described previously [21]. Briefly, formaldehyde (3.7%)-fixed cultures were treated with 1% hydrogen peroxide (10 min) followed by sequential incubation with blocking solution for 30 min, primary antibody overnight at 4°C, biotinylated secondary antibody for 2 h, and ABC reagents for 40 min. Color was developed with 3,3'-diaminobenzidine. For morphological analysis, the images were recorded with an inverted microscope (Nikon, Tokyo, Japan) connected to a charge-coupled device camera (DAGE-MTI, Michigan City, IN) operated with the MetaMorph software (Universal Imaging Corporation, Downingtown, PA). For visual counting of TH-IR neurons, nine representative areas per well of the 24-well plate were counted under the microscope at 100 × magnification by three individuals. The average of these scores was reported.

### Nitrite and tumor necrosis factor (TNF-α assays)

The production of NO was determined by measuring the accumulated levels of nitrite in the supernatant with Griess reagent and the release of TNF-α was measured with a rat TNF-α enzyme-linked immunosorbent assay kit from R and D System (Minneapolis, MN, USA), as described [23].

### Prostaglandin E_2 _(PGE_2_) production

PGE_2 _in supernatant was measured with a PGE_2 _EIA kit from Cayman (Ann Arbor, MI, USA) according to the manufacturer's instructions.

### Superoxide assay

The production of superoxide was determined by measuring the superoxide dismutase (SOD)-inhibitable reduction of the tetrazolium salt WST-1 [24,25]. Microglia-enriched cultures in 96-well culture plates were washed twice with HBSS without phenol red. Cultures were then incubated at 37°C for 30 min with vehicle control (water) or SN in HBSS (50 μl/well). Then, 50 μl of HBSS with and without SOD (50 U/ml, final concentration) was added to each well along with 50 μl of WST-1 (1 mM) in HBSS, and 50 μl of vehicle or LPS (10 ng/ml). To measure superoxide production induced by 0.2 uM MPP^+^, seven days-old mesencephalic neuron-glia cultures grown in 96-well plates were treated with SN in the presence or absence of MPP^+^, or vehicle in 150 μl of phenol red-free treatment medium. Four days after treatment, 50 μl of HBSS with and without SOD (50 U/ml, final concentration) was added to each well along with 50 μl of WST-1 (1 mM) in HBSS. Fifteen minutes later, absorbance at 450 nm was read with a SpectraMax Plus microplate spectrophotometer (Molecular Devices Corp, Sunnyvale, CA). The difference in absorbance observed in the presence or absence of SOD was considered to be the amount of superoxide produced, and results were expressed as percentage of vehicle-treated control cultures.

### Assay of intracellular reactive oxygen species

Intracellular oxidative stress was measured by DCFH Oxidation[26]. DCFH-DA enters cells passively and is deacetylated by esterase to nonfluorescent DCFH. DCFH reacts with ROS to form dichlorodifluorescein, the fluorescent product. DCFH-DA was dissolved in methanol at 10 mM and was diluted 500-fold in HBSS to give DCFH-DA at 20 μM. The cells were exposed to DCFH-DA for 1 h and then treated with HBSS containing the corresponding concentrations of LPS for 2 h. The fluorescence was read immediately at wavelengths of 485 nm for excitation and 530 nm for emission using a SpectraMax Gemini XS fluorescence microplate reader (Molecular Devices). The value subtracted by control group was viewed as the increase of intracellular ROS.

### Real time RT-PCR analysis

Real time quantitative PCR was performed as described previously [21], The relative differences between control and treatment groups were calculated and expressed as relative increases setting control as 100%. The sequence of the oligonucleotide primers from rats were: TNF-α: 5'-TCGTAGCAAACCACCAAGCA-3' and 5'-CCCTTGAAGAGAACCTGGGAGTA-3'; Inducible nitric oxide synthase (iNOS): 5'-ACATCAGGTCGGCCATCACT-3', and 5'-CGTACCGGATGAGCTGTGAATT-3'; Cyclo-oxygenases-2 (COX-2): 5'-CCAGCAG GCTCATACTGATAGGA-3' and 5'-GCAGGTCTGGGTCGAACTTG-3'; GAPDH: 5'-CCTGGAGAAACCTGCCAAGTAT-3' and 5'-AG CCCAGGATGCCCTTTAGT-3'.

### Confocal microscopy

Confocal analysis was performed as described previously [21]. HAPI cells seeded in dish at 5 × 10^4 ^cells/well were treated with LPS for 10 min in the absence or presence of SN (10^-14 ^M) pretreatment for 30 min. Cells were fixed with 3.7% paraformaldehyde in PBS for 10 min. After a washing with PBS, cells were incubated with rabbit polyclonal antibody against p47^*phox*^. Cells were then washed and incubated with FITC-conjugated goat anti-rabbit antibody. Focal planes spaced at 0.4-μm intervals were imaged with a Zeiss 510 laser scanning confocal microscope (63 × PlanApo 1.4 numerical aperture objective) equipped with LSM510 digital imaging software. The signal of p47^*phox *^(FITC-p47^*phox*^; green) and the merge view of cell morphology and p47^*phox *^(Phase plus FITC-p47^*phox*^) are shown. Scale bar, 50 μm.

### Membrane fractionation and western blot analysis

Membrane fractionation was performed as described (34). HAPI cells were lysed in hypotonic lysis buffer (1 mM EGTA, 1 mM EDTA, 10 mM β-lycerophosphate, 10 mM NaF, 1 mM sodium orthovanadate, 2 mM MgCl2, 10 mM DTT, 1 mM PMSF, and 10 ug/ml each leupeptin, aprotinin, and pepstatin A), incubated on ice for 30 min, and then subjected to Dounce homogenization (20~25 stokes, tight pestle A). The lysates were loaded onto sucrose in lysis buffer (final 0.5 M) and centrifuged at 1600 × g for 15 min, The supernatant above the sucrose gradient was centrifuged at 150,000 × g for 30 min. The resulting pellets were solubilized in 1% NP-40 hypotonic lysis buffer and used as membranous fraction. Equal amounts of protein (20 μg per lane) were separated by 4~12% Bis-Tris Nu-PAGE gel and transferred to polyvinylidene difluoride membranes (Novex, San Diego, CA). Membranes were blocked with 5% nonfat milk and incubated with rabbit anti-p47^*phox *^antibody (1: 2000 dilution) or mouse anti-gp91phox (1:1000 dilution) for 1 h at 25°C. Horseradish peroxidase-linked anti-rabbit or mouse IgG (1:3000 dilution) for 1 h at 25°C, ECL+Plus reagents (Amersham Biosciences Inc., Piscataway, NJ) was used as a detection system.

### Statistical analysis

The data are presented as mean ± SE. For multiple comparisons of groups, two-way ANOVA was used. Statistical significance of differences between groups was assessed using paired Student's t test, followed by Bonferroni correction using the JMP program (SAS Institute, Cary, NC, USA). A value of P < 0.05 was considered statistically significant.

## Results

### Effect of SN on LPS-induced DA neurotoxicity

**Figure 1:**
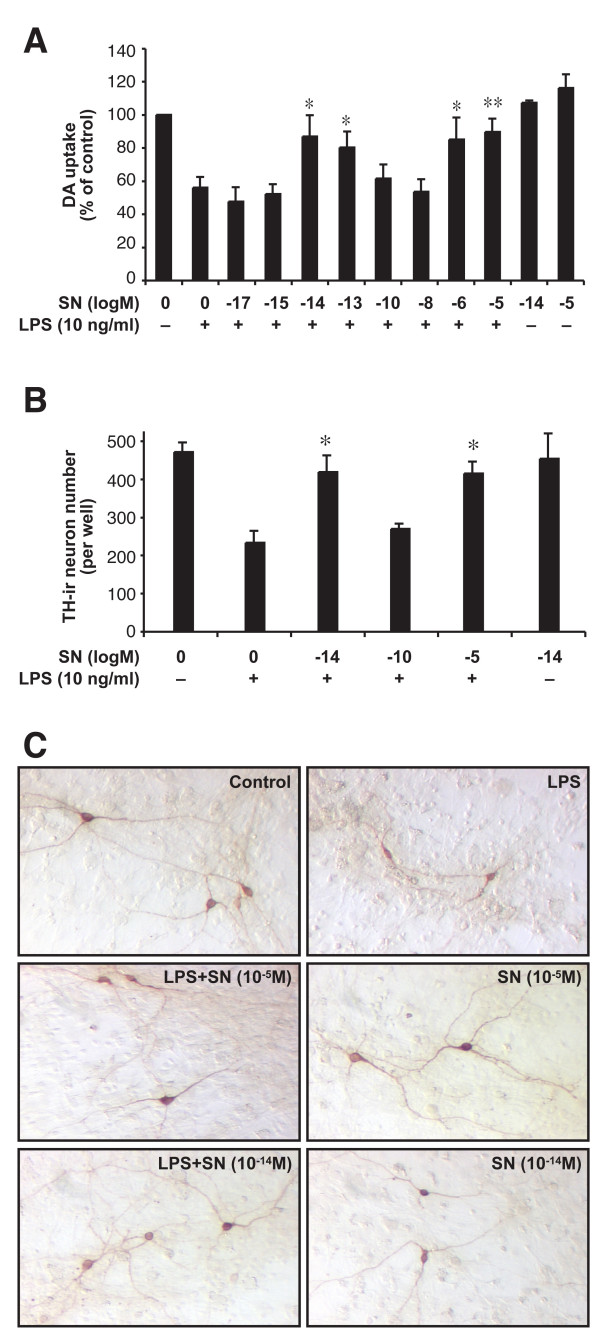
Both micromolar and sub-picomolar concentrations of SN are neuroprotective against LPS-induced neurotoxicity. Rat primary mesencephalic neuron-glia cultures seeded in a 24-well culture plate with 5 × 10^5 ^rat midbrain cells, then pretreated with SN (10^-17 ^to 10^-5 ^M) for 30 min before the addition of 10 ng/ml LPS. Eight days later, the LPS-induced dopaminergic neurotoxicity was quantified by the [^3^H]-DA uptake assay (*A*); by immunocytochemical analysis, including TH-IR neuron counts (*B*); and by representative pictures of immunostained sections (*C*). Results are expressed as percentage of vehicle-treated control cultures and represent the mean ± SE. for four (*A*) or three (*B, C*) independent experiments in triplicate. The mean absolute values of [^3^H]-DA uptake for vehicle-treated cultures range from 5000 to 7000 cpm. *P < 0.05, **P < 0.01 compared with the LPS-treated cultures.

We explored whether SN was neuroprotective at a wide range of concentrations (10^-17 ^to 10^-5 ^M). Mesencephalic neuron-glia cultures were pretreated with different concentrations of SN for 30 min before the addition of LPS for 7 days. The LPS-induced damage of DA neurons was then determined by [^3^H]-DA uptake assay (Fig. 1A) and by counting the number of TH-IR neurons (Fig. 1B). The [^3^H]-DA uptake assay showed that LPS treatment reduced the capacity of the cultures to take up DA to approximately 56% of the vehicle control. Interestingly, we found that both micromolar (10^-6^–10^-5 ^M) and sub-picomolar concentrations (10^-14^–10^-13 ^M) of SN could prevent this LPS-induced reduction. It is important to note that nanomolar concentrations of SN (10^-10^–10^-8 ^M) showed no significant protective effects (Fig. 1A). Identical protective effects were observed when we measured the number of viable TH-IR neurons with immunostaining (Fig. 1B). Morphological analysis revealed that LPS treatment not only decreased the number of TH-IR neurons, but also caused a loss of neuronal process, and these characteristics were also reversed by SN pretreatment in a concentration-dependent manner (Fig. 1C).

### SN pretreatment suppresses LPS-induced pro-inflammatory factors production and gene expression

**Figure 2:**
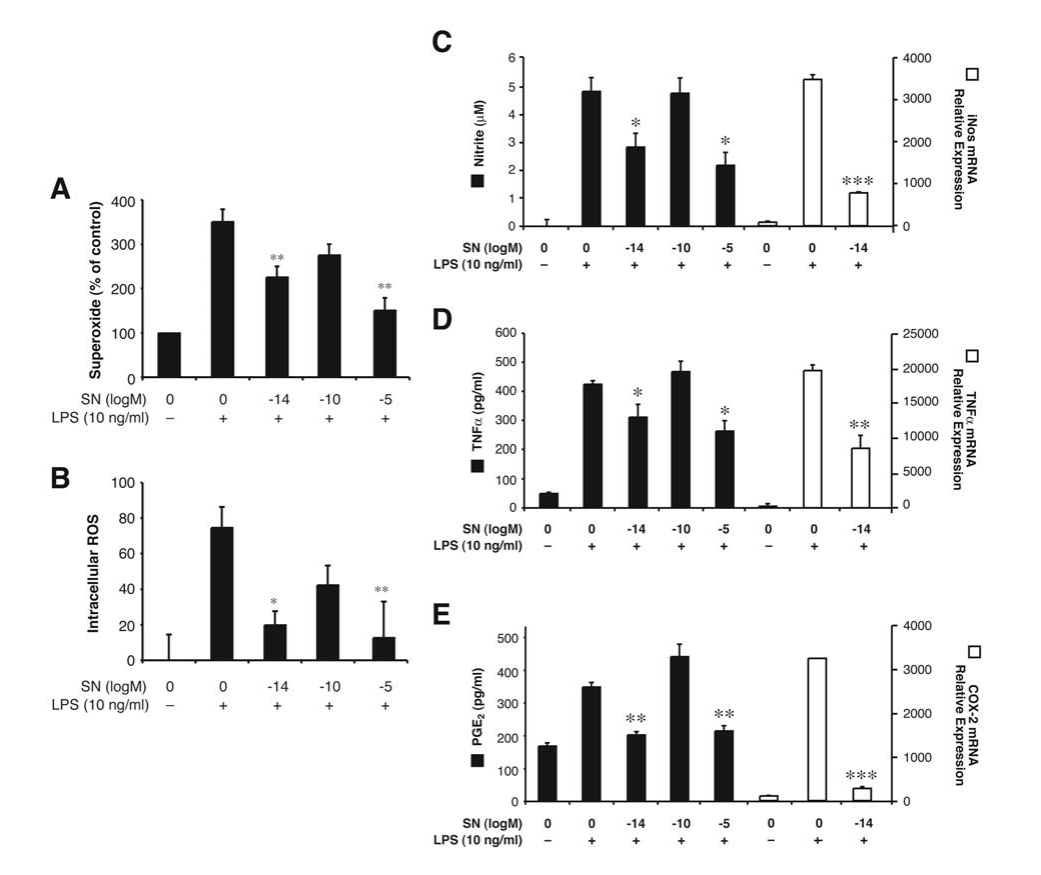
Effect of SN on LPS-induced production of pro-inflammatory factors and their mRNA expression. Enriched microglia cells were pretreated with vehicle or SN (10^-5^, 10^-10^, and 10^-14 ^M) for 30 min before LPS (10 ng/ml) stimulation. Effects of SN are shown on LPS-induced production of superoxide (*A*, expressed as % of control); and on intracellular ROS (*B*, expressed as absolute absorbance). Extracellular superoxide was measured as SOD-inhibitable reduction of WST-1, and intracellular ROS was determined by probe DCFH-DA. Supernatant was collected at 24 h for nitrite assay (*C*), at 3 h for TNF-α assay (*D*), and at 24 h for PGE_2 _assay (*E*). RNA were extracted at 3 h after LPS stimulation; the effect of SN, at sub-picomolar concentrations, on LPS-induced iNOS, TNF-α, and COX-2 mRNA expression (*C-E *respectively, open bars) are shown. Results are expressed as mean ± SE for three independent experiments performed in triplicate. *P < 0.05, **P < 0.01 compared with the LPS-treated cultures.

We have previously reported that microglia play a critical role in mediating LPS-induced neurotoxicity [27]. LPS can activate microglia to produce an array of pro-inflammatory mediators, and among them ROS is the key factor mediating inflammation-related neurotoxicity [28]. We first tested the effect of SN on the LPS-induced ROS generation, including extracellular superoxide and intracellular ROS in microglia-enriched cultures. As shown in Figs. 2A and 2B, SN at 10^-5 ^M and 10^-14 ^M, but not 10^-10 ^M, significantly attenuated LPS activation-mediated superoxide production and levels of intracellular ROS. We further investigated other pro-inflammatory mediators induced by activated microglia, including TNF-α, PGE_2_, and nitrite, all of which have been previously reported to be neurotoxic [2]. Enriched microglia were pretreated with vehicle or SN for 30 min before the addition of LPS. Three hours after LPS stimulation, RNA was extracted and real-time PCR assay was performed. As shown in Fig. 2C, both micromolar and sub-picomolar concentrations of SN significantly inhibited nitric oxide production and iNOS mRNA expression and TNF-α protein production and mRNA expression (Fig. 2D), as well as PGE_2 _production and COX-2 mRNA expression (Fig. 2E) in enriched microglia, while SN at 10^-10 ^M showed no effect (Fig. 2). Since both micromolar and sub-picomolar concentrations of SN show equal potency in their neuroprotective effects by inhibiting microglia activity, we focused only on sub-picomolar concentration (10^-14^M) for further study.

### SN also suppresses MPP^+^-induced neurotoxicity by inhibition of microglia activation

**Figure 3:**
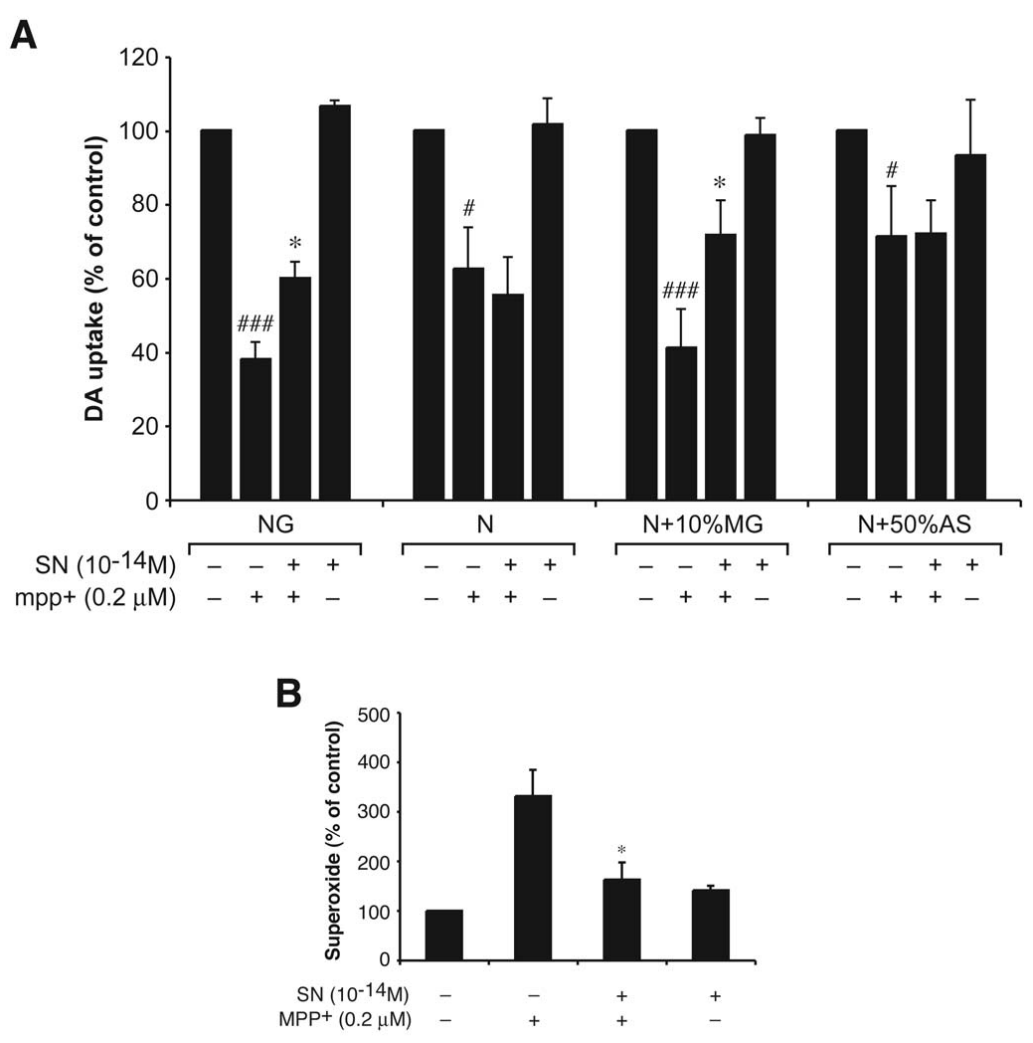
SN protects against MPP^+^-elicited DA neurodegeneration through microglia. SN (10^-14 ^M) or MPP^+ ^(0.2 μM) were added to the following types of cell cultures: (NG): original neuron-glial cultures; (N): neuron-enriched cultures; (N+10%MG): cultures reconstituted by adding 10% of microglia to the neuron-enriched cultures; (N+50%AS): cultures reconstituted by adding 50% of astroglia to the neuron-enriched cultures. Two and 4 days after MPP^+ ^treatment, SN (10^-14 ^M) was added again to the SN-treated cultures. On day 8, the MPP^+^-induced dopaminergic neurotoxicity was quantified by the [^3^H]-DA uptake assay (A), and on day 4, the release of superoxide was determined as described in Materials and methods section. Results are expressed as percentage of the vehicle-treated control cultures and represent the mean ± SE. for three independent experiments performed in triplicate. *P < 0.05, compared with MPP^+ ^treated cultures. # *P *< 0.05, ## *P *< 0.01 compared with the vehicle-treated control cultures.

1-Methyl-4-phenylpyridinium (MPP^+^), the active metabolite of MPTP, is known to damage DA neurons directly. Since MPTP/MPP^+ ^has been widely used in rodent PD model, we sought to determine whether SN would inhibit MPP^+^-induced neurotoxicity. Neuron-glial cultures were pretreated with SN for 30 min, then incubated with 0.2 μM MPP^+ ^for seven days, and neurotoxicity was assayed by DA uptake. Addition of MPP^+ ^resulted in a decrease in DA uptake (reduced to 38% of control), and this toxicity was partially but significantly reversed by pretreatment of these cells with SN (recovered to 60% of control) (Fig. 3A – NG). This indicates that sub-picomolar concentration of SN protects against the neurotoxic effects of MPP^+^, but there is a level of toxicity mediated by the direct toxic effect by MPP^+ ^on neurons that is independent of the protective effects of SN.

We then sought to determine the cellular target of SN-mediated neuroprotection. Results showed that SN had no effect on the toxic effects of MPP^+ ^in neuron-enriched cultures (Fig. 3A – N), demonstrating that SN does not function by directly protecting neurons from MPP^+^-mediated toxicity. We then investigated if glial cells were the target for the neuroprotective activity of SN by reconstituting neuron-enriched cultures with either 10% purified microglia (Fig. 3A – N + 10% MG) or 50% astroglia (Fig. 3A – N+50% AS). The percentage of glial cells chosen for reconstitution was based on the fact that our standard mesencephalic neuron-glia cultures contain ~10% microglia and ~50% astrocytes in addition to DA and other neurons. We found that while SN is not able to protect DA neurons in neuron-astroglia reconstituted cultures, SN was able to reduce neurotoxicity in neuron-microglia reconstituted cultures to a level similar to that seen in the original neuron-glia cultures. This result indicates that there is an additional, indirect microglia-mediated neurotoxicity by MPP^+ ^through reactive microgliosis, a process initiated when the death or damage of neurons triggers further activation of microglia [27].

Previous work from our laboratory and others have indicated that oxidative stress plays a very important role in the progressive neurodegeneration, and MPTP/MPP^+^-induced reactive microgliosis has been clearly linked with microglial activation and closely associated with increased production of oxygen free radicals [27,29]. Therefore, we sought to determine whether SN is able to reduce MPP^+^-induced ROS production. Rat mesencephalic neuron-glia cultures were pretreated with SN (10^-14 ^M) before the addition of MPP^+^. Release of superoxide from activated microglia was then determined on day 4 after treatment. As shown in Fig. 3B, treatment of cultures with SN significantly inhibited MPP^+^-induced superoxide production. Interestingly, we found that while significant superoxide production was detected following MPP^+^-induced reactive microgliosis, the production of other inflammatory mediators, including TNF-α and nitrite, were not detectable at any time during the 7-day MPP^+ ^treatment in neuron-glial cultures (data not shown). Taken together, these results demonstrated that SN can inhibit ROS production induced either directly by LPS stimulation or indirectly by MPP^+^-mediated reactive microgliosis, and lend further evidence to the idea that superoxide production, rather than TNF-α or nitrite production, is the mechanism of neurotoxicity in reactive microgliosis.

### PHOX is essential for SN mediated neuroprotection

**Figure 4:**
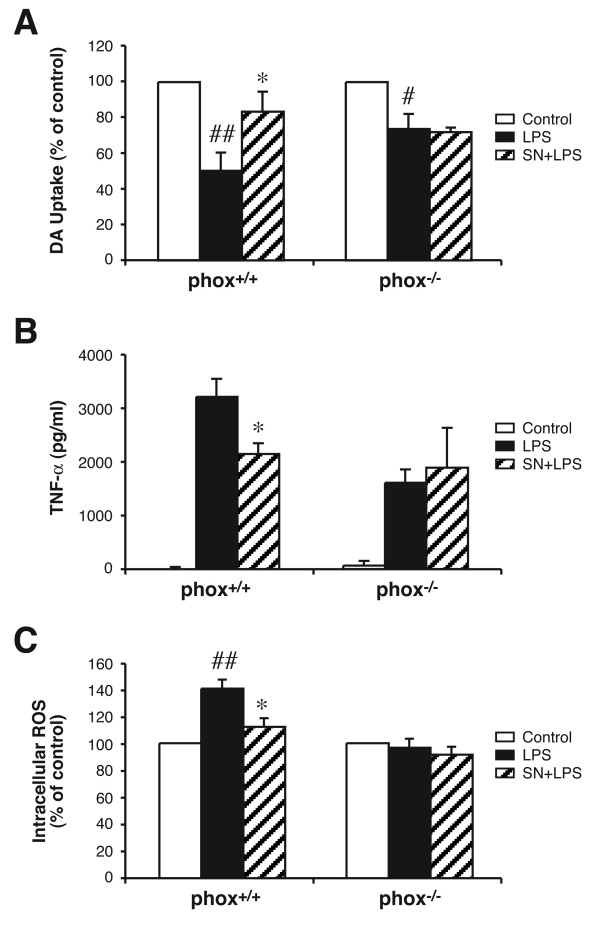
Microglial PHOX is critical for sub-picomolar SN neuroprotection. PHOX^+/+ ^and PHOX^-/- ^mouse neuron-glia cultures were pretreated with vehicle or SN (10^-14 ^M) for 30 min, followed by LPS treatment. Neurotoxicity was assessed by measuring DA uptake (*A*), TNF-α production (*B*) and intracelluar ROS (*C*), respectively. Results are expressed as % of the control culture (*A *and *C*) and as pg/ml (*B*), and represent the mean ± SE for 3 individual experiments performed in triplicate in each experiment. *P < 0.05 compared with LPS culture. # *P *< 0.05, ## *P *< 0.01 compared with the vehicle-treated control cultures.

Previous reports from our laboratory indicated that PHOX is the major enzyme in microglia that produces extracellular superoxide anions and is a major contributor to the increase of the intracellular ROS, which in turn enhances TNF-α production [28]. To further study the role of ROS in SN-mediated neuroprotection, neuron-glia cultures were prepared from PHOX-deficient (PHOX^-/-^) and wild-type (PHOX^+/+^) mice. As shown in Fig. 4A, LPS treatment of cultures prepared from PHOX^+/+ ^mice reduced [^3^H]-DA uptake by 50%, while SN (10^-14 ^M) significantly attenuated the decrease. In contrast, LPS treatment reduced the uptake capacity by 27% in PHOX^-/- ^mice, but SN failed to show any protective effect. LPS-induced TNF-α production in PHOX^-/- ^mice is only half of that in PHOX^+/+ ^mice, and while SN was able to significantly reduce TNF-α production in PHOX^+/+ ^mice, no significant change was seen in PHOX^-/- ^mice (Fig. 4B). Similarly, SN at 10^-14 ^M significantly inhibited intracellular ROS production in PHOX^+/+ ^mice, but not in PHOX^-/- ^mice (Fig. 4C). These results strongly support the hypothesis that inhibition of ROS production and subsequently TNF-α production is critical to the neuroprotective effect of SN.

**Figure 5:**
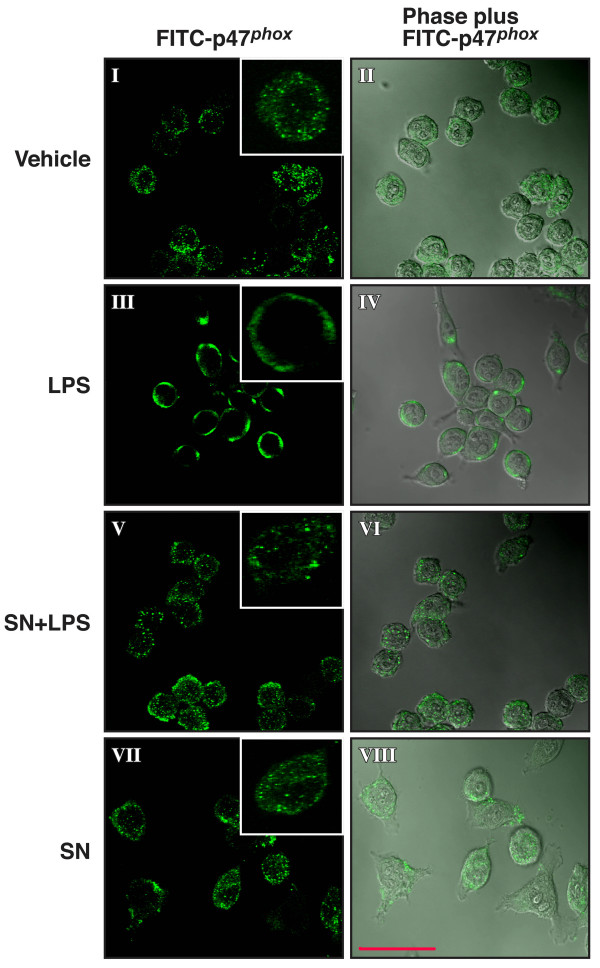
Immunofluorescence and confocal microscopical analysis of p47^*phox *^localization in LPS-stimulated microlgia cells. HAPI cells were treated with LPS for 10 min in the absence or presence of SN pretreatment for 0.5 h. Cells were immunostained with a rabbit polyclonal antibody against p47^*phox*^, then washed and incubated with FITC-conjugated goat anti-rabbit antibody. The signal of p47^*phox *^(FITC-p47^*phox*^; on left) and the merge view of cell morphology and p47^*phox *^(Phase plus FITC-p47^*phox*^, on right) are shown. The inset shown in the right corner of each treatment condition shows the location of FITC-p47^*phox *^in a single, randomly selected cell. Focal planes spaced at 0.4-μm intervals were imaged with a Zeiss 510 laser scanning confocal microscope (63 × PlanApo 1.4 numerical aperture objective) equipped with LSM510 digital imaging software. Three adjacent focal planes were averaged using Metamorph software. The signal of p47^*phox *^(FITC-p47^*phox*^; green) and the merge view of cell morphology and p47^*phox *^(Phase plus FITC-p47^*phox*^) are shown. Scale bar, 50 μm.

**Figure 6:**
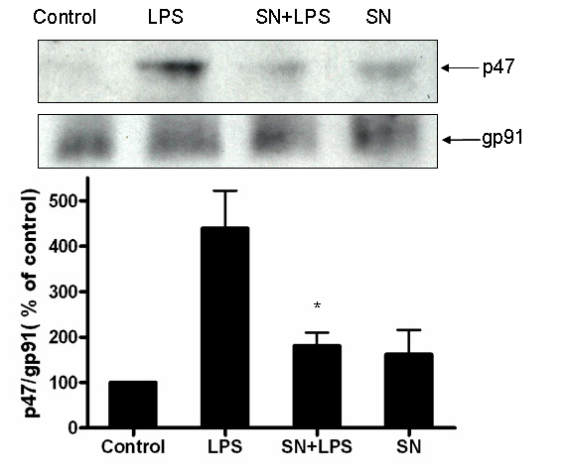
**Membrane fraction analysis of the effect of SN on cytosolic p47**^*phox*^**protein translocation**. HAPI cells were pretreated with vehicle or SN (10^-14 ^M) for 30 min, followed by LPS treatment for 10 min. Membrane fractions were isolated to perform western blot analysis, using gp91^*phox *^as an internal membrane control. Each experiment was performed three times. *P < 0.05, compared with the LPS-treated cultures.

Previous studies have shown that activation of PHOX activity requires that the cytosolic component of the PHOX enzyme, p47^*phox*^, become phosphorylated and translocate along with p67^*phox*^, and p40^*phox*^, to the plasma membrane where it associates with cytochrome b558 and assembles into an active enzyme [30]. Since SN significantly inhibits superoxide production and fails to protect DA neuron damage induced by LPS in PHOX^-/- ^mice, we sought to determine if SN inhibits PHOX activation by preventing the p47^*phox *^translocation from cytosol to membrane following LPS stimulation in HAPI cells, a microglial cell line from rat. Using confocal microscopy, we observed that in un-stimulated cells (Fig. 5, panel I and II) or in cells treated with SN alone (Fig. 5, panel VII and VIII), p47^*phox *^was found primarily localized in the cytosol. In contrast, ten minutes after LPS stimulation, we detected virtually all the p47^*phox *^clustered at the membrane (Fig. 5, panel III and IV). However, the addition of 10^-14 ^M SN significantly prevented this LPS-induced p47^*phox *^translocation (Fig. 5, panel V and VI). Consistent with the results of the confocal microscopy study, western blot assay clearly showed an increase in the immunoreactivity of p47^*phox *^in the membrane fraction of HAPI cells 10 min after LPS treatment and this increase in the immunoreactivity was significantly blocked in the presence of SN at 10^-14 ^M. LPS did not show any effects on gp91 protein expression at this time point (Fig. 6). Therefore, one mechanism by which SN inhibits superoxide production in microglial cells is through inhibition of p47^*phox *^translocation to the cell membrane following LPS stimulation.

## Discussion

The main findings in this study are the elucidation of anti-inflammatory and neuroprotective effects of SN at both the cellular and molecular level. Using both LPS and MPP^+^-mediated PD models, we are the first to demonstrate that SN could be effective in diminishing inflammation-induced neurodegeneration at both micro- and sub-picomolar concentrations in primary midbrain neuron-glia cultures. Mechanistic studies revealed that inhibition of microglial PHOX activity is the target for SN-mediated neuroprotection in both LPS- and MPP^+^-induced DA neurotoxicity. The mechanism underlying SN-mediated inhibition of PHOX activity occurs at least in part through the inhibition of the translocation of PHOX cytosolic component p47^*phox *^to the plasma membrane, a key event required for extracellular ROS production [31]. We are currently investigating the effects of SN on the activity of the other subunits of the PHOX enzyme, including p67^*phox*^, p40^*phox*^, gp91^*phox*^, p22^*phox *^and Rac2. The inhibition of PHOX leads to a subsequent reduction in the production of other pro-inflammatory mediators, such as intracellular ROS, TNF-α, NO and PGE_2_. Our studies suggest that reduction in PHOX activity by SN at the site of inflammation diminishes host tissue damage, thereby underlying the neuroprotective effect of SN for both LPS- and MPP^+^-induced DA neurotoxicity.

Although SN has been used clinically as an anti-inflammatory agent in several inflammation-related diseases [10,11], so far only a few studies have addressed the immunomodulatory mechanism of this herbal medicine. For example, it has been reported that SN inhibits the production of pro-inflammatory mediators, such as TNF-α, IL-1, PGE_2_, leukotriene C4 and NO from macrophages [15,32,33]. Our observations have not only extended the above findings to microglia, but more importantly, our studies indicate that the reduction in the release of these pro-inflammatory factors by SN could be due to the inhibition of superoxide production through the inhibition of microglial PHOX activity. Our earlier studies established that inhibition of microglial production of superoxide was most effective in protecting neurons, indicating that superoxide was a dominant degenerative factor for the DA neurons in the culture [18]. Our evidence that SN significantly inhibits the production of superoxide induced by either LPS or MPP^+^, as well as the observation that ROS production is the only inflammatory mediator that can be detected after exposure of neuron-glial cultures to MPP^+^, led us to examine the role of PHOX in neurotoxicity in greater detail by using PHOX-deficient mutant mice. Our findings that sub-picomolar SN could significantly lessen the LPS-induced DA uptake reduction in wild-type mice, has no significant protective effect in PHOX^-/- ^mice (Fig. 4A) strongly support the conclusion that PHOX activity is critical to SN-mediated DA neuroprotection. Moreover, the production of intracellular ROS and release of TNF-α are both reduced in PHOX^-/- ^mice, suggesting PHOX can indirectly regulate intracellular ROS concentration and ultimately the production of pro-inflammatory mediators. We can not rule out the possibility that cells other than microglia are producing superoxide, and that PHOX is not the only enzyme which may play a role in ROS production. However, only microglia express LPS receptors and are activated by LPS, and therefore no other cell is likely to produce ROS upon stimulation with LPS. In addition, no extracellular superoxide production has been detected in cultures from PHOX^-/- ^mice (Qin et al., 2004), suggesting that PHOX is the only enzyme involved in superoxide production in these cultures. It has been found that an increase of intracellular ROS can intensify the activation of NF-kB, which leads to higher TNF-α and PGE_2 _production [2,28]. In addition, it has been reported that PHOX inhibitors prevent LPS/IFNγ-induced degradation of IkB and, thus, inhibit the activation of NF-kB [34]. These data are consistent with the notion that PHOX plays a central role in both inflammation-induced neurotoxicity, as well as in the regulation of the inflammatory response by microglial cells, and that SN can inhibit these inflammatory responses by inhibiting PHOX activity.

The bimodal dose response of SN in the inhibition of LPS-induced superoxide production and neuroprotection adds SN to the list of compounds which show the same pattern of responsiveness, including dynorphins [35], enkephalins [36], endorphin [37], pituitary adenylate cyclase-activating polypeptide (4–6) [38]. Currently it is still not clear how both micro- and sub-picomolar concentrations of SN and the other compounds inhibit PHOX activation. To further understand how SN inhibits PHOX activity, we performed the translocation experiment using confocal microscopy. We found that the translocation of the p47^*phox *^subunit from the cytosol to the plasma membrane following LPS stimulation was inhibited by SN. As the activation of a functional PHOX enzyme requires the phorsphorylation of p47^*phox *^followed by the translocation of the p47^*phox*^, p67^*phox*^, and p40^*phox *^complex to the plasma membrane, our results suggest that SN works on one of the earliest stages of PHOX activation to inhibit the assembly of the PHOX enzyme complex. Presently the molecular mechanisms by which SN leads to the inhibition of p47^*phox *^translocation are under investigation.

It is interesting to note that SN has protective effects in both the LPS and the MPP^+ ^model of PD, although the mode of action for these two agents to produce neurotoxicity is different. In the LPS model, it is clear that direct activation of microglia by LPS leads to the activation of PHOX, which results in the production of superoxide that mediates the neurotoxicity, and that direct inhibition of PHOX by SN in microglia can prevent this destruction. However, even though MPP^+ ^directly targets DA neurons to produce toxicity, SN still shows a significant protective effect in these cultures as well. This is most likely due to the fact that a significant portion of the MPP^+^-mediated neurotoxicity requires the presence of microglial cells, suggesting that MPP^+ ^works both directly to kill a subset of DA neurons, but also indirectly to activate microglia through reactive microgliosis. Recent evidence indicates that neuronal death or damage triggers activation of microglia through either release of soluble factors or loss of cell-cell contact inhibition between neurons and microglia [27,39]. This activation of microglia by dying neurons then continuously damages the remaining DA neurons, and creates a self-propelling inflammatory cycle, which may underlie the progressive nature of neurodegenerative diseases such as PD. Reports from our laboratory and others have indicated that MPP^+ ^can cause reactive microgliosis, and that oxidative stress is involved in MPTP/MPP^+ ^-induced neurotoxicity [27,40]. Our observation that MPP^+^-mediated neurotoxicity is significantly less in PHOX^-/- ^animals [41], and that SN does not protect DA neurons from MPP^+^-mediated toxicity in neuron-enriched or neuron-astroglial cultures (Fig. 3), supports the notion that SN protects MPP^+^-induced neurotoxicity mainly through the inhibition of ROS production, which in turn slows down the self-propelling cycle and prevents further neuronal death. In addition, although LPS and MPP^+ ^work in different ways to produce neurotoxicity, the death of neurons induced by both LPS and MPP^+ ^will further activate microglia through the process of reactive microgliosis, in which superoxide plays a critical role.

## Conclusion

In summary, this study is the first report indicating that SN is a potent anti-inflammatory and neuroprotective agent that acts through inhibition of microglial PHOX-generated superoxide by inhibiting the translocation of p47^*phox *^to the plasma membrane. We have previously reported that several morphinan compounds, such as naloxone, exert their neuroprotective effects independent of the conventional opioid receptors [18,22], because the dextrorotatory form of this compound, which binds poorly to the opioid receptors, is equipotent with its levorotatory isomer. SN, being a natural dextrorotatory form of morphinan that can be easily extracted and purified from its herb plant, is an ideal alternative for other synthesized dextrorotatory form morphinan compounds since the dextrorotatory isomers are extremely expensive to fabricate. In view of its potent anti-inflammatory and neuroprotective effects, its safe record of clinical usage to treat rheumatoid arthritis, and its relatively low cost, SN is an ideal candidate for animal and clinical trial studies to evaluate its therapeutic potential as a neuroprotective agent for PD and other inflammation-related neurodegenerative diseases.

## Competing interests

The author(s) declare that they have no competing interests.

## Authors' contributions

LQ performed experiments and drafted the manuscript. ZLX performed the statistical analysis. WZ participated in the experiments. BW carried out the immunostaining assay. JSH and PMF conceived the study and its design and helped to draft the manuscript. All authors read and approved the final manuscript.
